# Statistical shape modelling of the human lumbar spine: a scoping review characterising the research landscape and potential clinical applications

**DOI:** 10.3389/fbioe.2026.1884249

**Published:** 2026-09-09

**Authors:** Gordon Wai, Kay A. Raftery, Francis H. V. Lali, Liliana Rodrigues, Karen M. Knapp, Judith R. Meakin, Nicolas Newell

**Affiliations:** 1 Department of Bioengineering, Imperial College London, London, United Kingdom; 2 Faculty of Medicine and Dentistry, Queen Mary University of London, United Kingdom; 3 Department of Health and Care Professions, Faculty of Health and Life Sciences, Medical Imaging, University of Exeter, United Kingdom; 4 Department of Physics and Astronomy, Faculty of Environment, Science and Economy, University of Exeter, United Kingdom

**Keywords:** lumbar spine, scoping review, statistical shape and appearance modeling, statistical shape modeling (SSM), systematic review

## Abstract

**Objective:**

A systematic scoping review was conducted to characterise the current research landscape of statistical shape modelling of the lumbar spine, focusing on the proposed clinical applications of the method.

**Methods:**

MEDLINE, Embase, IEEE Xplore, and Scopus were searched for sources published before 17 June 2026. Sources were screened against predefined inclusion and exclusion criteria and data were extracted using a structured form in Covidence.

**Results:**

2,930 records were identified for review. Following duplicate removal and screening, 85 studies remained for data extraction. Most studies were exploratory (57%) or descriptive (14%) in nature. The vertebral body was the most frequently isolated region, with L5 often excluded due to its anatomical distinctiveness. Reporting of population demographics and dataset composition was highly inconsistent, highlighting substantial heterogeneity in both methodology and population selection.

**Conclusion:**

The use of lumbar spine statistical shape models (SSMs) has been predominantly exploratory or descriptive. Whilst prognostic, diagnostic, and therapeutic applications have been proposed, none of the reviewed studies demonstrated a direct change to clinical practice or impact on patient care. No consensus exists on optimal approaches to multi-level modelling, vertebral level pooling, or anatomical boundary definition, and training datasets are predominantly drawn from Western, female-skewed populations. In the short term, SSMs are well suited to exploratory analyses of clinical populations, revealing patterns not captured by conventional measures. Progress will benefit from training data aligned to defined clinical questions, adequate sample sizes and demographic breadth, and consistent reporting of validation metrics such as compactness, generalisability and specificity. This will enable development towards a technology that can lead to meaningful clinical translation.

## 1 Introduction

Statistical shape models (SSMs) are geometric models used in medical image analysis to quantify and characterise anatomical shape variation across populations (Lindner, 2017). Central to SSM construction is the establishment of shape correspondence, whereby the geometry of each shape is represented in a consistent manner across all subjects, from which statistical analysis of shape variation can subsequently be performed (Heimann and Meinzer, 2009). The classical and most common methodology is to apply Principal Component Analysis (PCA) to a set of corresponding landmark points describing the geometry of each shape (Cootes and Taylor, 2001). However, SSMs comprise a methodologically heterogeneous family of approaches, varying in the strategies employed to establish correspondence, the statistical frameworks applied, and the procedures used to validate model performance (Cai et al., 2024)

The lumbar spine is a region of considerable clinical and research interest, with low back pain constituting the leading cause of years lived with disability globally, affecting 619 million people in 2020 and projected to rise to 843 million by 2050 (Ferreira et al., 2023). SSMs have been applied extensively in this context to study vertebral morphology and shape variation. The spine is multi-level and mechanically coupled, with local vertebral and disc geometry covarying with overall sagittal alignment. SSMs are used to characterise this combined variability across levels and summarise it in interpretable modes for cohort comparison (Hollenbeck et al., 2018). Population-based models and atlases have quantified shape and alignment in healthy and pathological spines, providing a basis for comparative analyses across demographics and conditions (Sciortino et al., 2022). Within imaging workflows, SSMs also act as anatomical priors that support segmentation and registration in challenging spinal data (Rasoulian et al., 2013).

Despite the number of published SSMs, many function as stand-alone research tools designed for specific populations. A review to assess progress and consider their potential clinical applications would be useful to focus future research efforts. Extension of this review beyond the lumbar spine was not pursued, as differences in correspondence strategies, training requirements, and validation benchmarks between anatomical structures preclude meaningful comparison across regions. A narrative review of SSMs across orthopaedic applications has previously been conducted (Cai et al., 2024); however, anatomy-specific scoping reviews, such as that performed for the human hip joint by Johnson et al. are able to address more focused research questions with greater methodological rigour (Johnson et al., 2023). No such review currently exists for the lumbar spine.

This systematic scoping review aims to map the development and application of published lumbar spine statistical shape models (SSMs). Specifically, it examines how lumbar spine SSMs have been utilised in the existing literature, the clinical and research applications for which they have been developed, how the unique challenges associated with constructing lumbar spine SSMs have been addressed, and the populations that have been studied.

## 2 Methods

This review was performed under the guidance of the Imperial College London Library Service’s systematic review guide (Imperial College London, 2025). Before undertaking the review, a search for registered reviews of the term “statistical shape modelling”, “bone shape modelling”, and “statistical shape” were performed in Google Scholar and PROSPERO (NIHR, 2025). Although scoping reviews of the hip were identified (van Buuren et al., 2021; Johnson et al., 2023), and one protocol addressing vertebral bone shape modelling and its association with osteoporotic-related fractures was found (Rodrigues et al., 2023), no review was found to have examined the broader development and applications of lumbar spine statistical shape models.

The review has been reported using the Preferred Reporting Items for Systematic Reviews and Meta-Analyses extension for Scoping Reviews (PRISMA-ScR) (Tricco et al., 2018). *A priori* protocol was not published.

### 2.1 Inclusion and exclusion criteria

This report considers any human SSM of the lumbar spine presented in journal articles and conference papers. The different anatomical shapes of the vertebrae and spinal curvature between the lumbar, thoracic and cervical segments of the spine mean it would not be useful to compare the entire spine in a single review. Inclusion and exclusion criteria are presented in Table 1.

**TABLE 1:** Inclusion and exclusion criteria for the scoping review.

Inclusion	Exclusion
SSMs of the human lumbar spine	Models of the sacrum, cervical or thoracic spine (that do not include the lumbar spine)
Active Shape Models (ASMs)	Models of just the Intervertebral Discs (IVDs)
Active Appearance Models (AAMs)	Models including extraspinal anatomy (e.g., trunk surface or ribs)
​	Statistical alignment models (with no shape analysis)
​	Animal models
​	Conference abstracts and posters
​	Non-English papers

Studies were eligible for inclusion if they reported the development or application of an SSM of the human lumbar spine. Active Shape Models (ASMs) and Active Appearance Models (AAMs) were included as they represent established methodological variants within the SSM family.

Studies modelling the sacrum, cervical or thoracic spine without inclusion of the lumbar spine, those restricted exclusively to the intervertebral discs, and those incorporating extraspinal anatomy such as the trunk surface or ribcage were excluded for the anatomical reasons outlined in the introduction. Statistical alignment models without shape analysis were excluded, as these techniques capture gross spinal curvature through landmark-based spline fitting rather than characterising shape variation, representing a substantially different methodological approach. Animal models were excluded given the clinical translational focus of this review. Conference abstracts and poster presentations were excluded as the available methodology reporting was insufficient to permit quality assessment. Non-English language papers were excluded due to resource constraints.

### 2.2 Search strategy

An initial search of MEDLINE (Ovid) was performed to identify appropriate papers for inclusion. Citation searching was then undertaken in consultation with health sciences and bioengineering librarians at Imperial College London to identify relevant missed papers and refine the search strategy. A pilot test of 10 randomly selected papers was conducted until no further adjustments were necessary. The final strategy for MEDLINE (Ovid) was then adapted for use with other databases. The following databases were searched: MEDLINE (Ovid), Embase (Ovid), IEEE Xplore, and Scopus. Full details of the search strategy are provided in the Supplementary Material.

Papers published prior to the final MEDLINE (Ovid) search on the 17 June 2026 were included. No limit was specified for the earliest inclusion date as SSMs were first described by Cootes et al., in 1992, which was deemed recent enough for all potential studies to be considered (Cootes et al., 1992).

### 2.3 Study selection

A single reviewer was used for all stages of study selection and data extraction. Search results were imported into Covidence (Veritas Health Innovation, 2024) where duplicates were automatically removed (Veritas Health Innovation, 2024). Titles and abstracts were then screened against the inclusion and exclusion criteria. The remaining papers then underwent full-text screening. At this stage, any duplicates that had not been automatically removed were manually removed. Papers referencing previously published SSMs were merged with the original to avoid duplicate reporting.

### 2.4 Data extraction

A data extraction form was created in Covidence, based on the review questions outlined in Section 1. A summary of the full data extraction form may be found in the Supplementary Material. The form was used by a single reviewer for each included study.

Firstly, general characteristics of each study were captured, such as the publication date and journal. To address how SSMs of the lumbar spine have been utilised in existing literature, six categories (exploratory, descriptive, diagnostic, prognostic, therapeutic, other) were defined to describe the overall aims of the model in the study (Table 2). These categories were adapted from a scoping review of SSM in the hip by Johnson et al. (2023).

**TABLE 2:** Description of categories assigned to each study included in the scoping review.

Category	Definition
Exploratory	Study in which the primary contribution is the development or refinement of SSM methodology, including proof-of-concept models and automated segmentation or registration pipelines, where the model itself constitutes the primary output
Descriptive	Study in which an SSM is employed as an analytical tool to characterise morphological variation within a population, where the population-level findings constitute the primary output
Diagnostic	Study uses an SSM to identify conditions or extract key variables to aid diagnosis (e.g., Cobb angle)
Prognostic	Study uses an SSM to identify indicators that may predict disease progression or clinical outcomes
Therapeutic	Study uses an SSM to guide treatment planning or intervention strategies
Other	Does not align with the categories above

Studies that proposed automated segmentation or landmark registration as the sole application of SSMs were classified under the category of ‘exploratory,’ unless further analysis demonstrated clinical or diagnostic utility. This approach was used to emphasise novel contributions to the field. For example, investigations involving the reconstruction of spinal pathologies and validated only using segmentation error metrics were considered ‘exploratory’, whereas those of similar application, that also evaluated the accuracy of clinical measures, were categorised as ‘diagnostic’. A one-sentence summary of the model’s aims was also recorded.

To assess SSM creation methods, several multiple-choice selections were created to record the landmarking method (‘Manual’, ‘Semi-automatic’, ‘Automatic’, or ‘Other’; the latter capturing studies that did not require landmark-based correspondence or did not report their approach), dimensionality reduction method (‘Classical PCA’, ‘Kernel PCA’, ‘Single value decomposition’, etc.), and which region of the vertebra was modelled (‘Whole vertebra’, ‘Vertebral body’, ‘Pedicle’, etc.). The number of models created was also recorded.

The spine holds unique challenges with regard to SSM as it is a series of highly similar adjacent vertebrae (Chu et al., 2015). This raises key questions such as: Should each vertebral level be treated as a distinct structure or as variations of the same bone? How should their alignment and spatial relationships be accounted for during modelling? To capture this, multiple-choice options were created to capture whether vertebral levels were pooled, and whether the models were of a single vertebral level or multi-level. Studies that presented an SSM composed of multiple separate models for each vertebral level were considered to have presented a single SSM.

To assess what populations have been studied, the World Health Organisation (WHO) region^1^ of each training dataset was recorded, along with imaging modality, gender split, and age demographics (World Health Organisation, 2026). The mean, standard deviation, and range were recorded. The source of each model was also logged to identify whether the same population had been used to develop multiple models. The size of the training dataset was also recorded. Finally, to assess which pathologies have been studied with an SSM, a multiple-choice option was created to record the pathologies researched. If no pathology was stated, the members of the dataset were assumed to be ‘healthy’.

If a distinct population was used to test the SSM, the same demographic and dataset characteristics were recorded for the testing cohort.

### 2.5 Data synthesis

Following data extraction, the extracted data were exported to a spreadsheet for tabulation and figure generation. At this stage, duplicate training and testing populations were manually identified. Overlapping populations across studies were also noted, and for the purposes of synthesis, all were treated as distinct datasets.

## 3 Results

**FIGURE 1:**
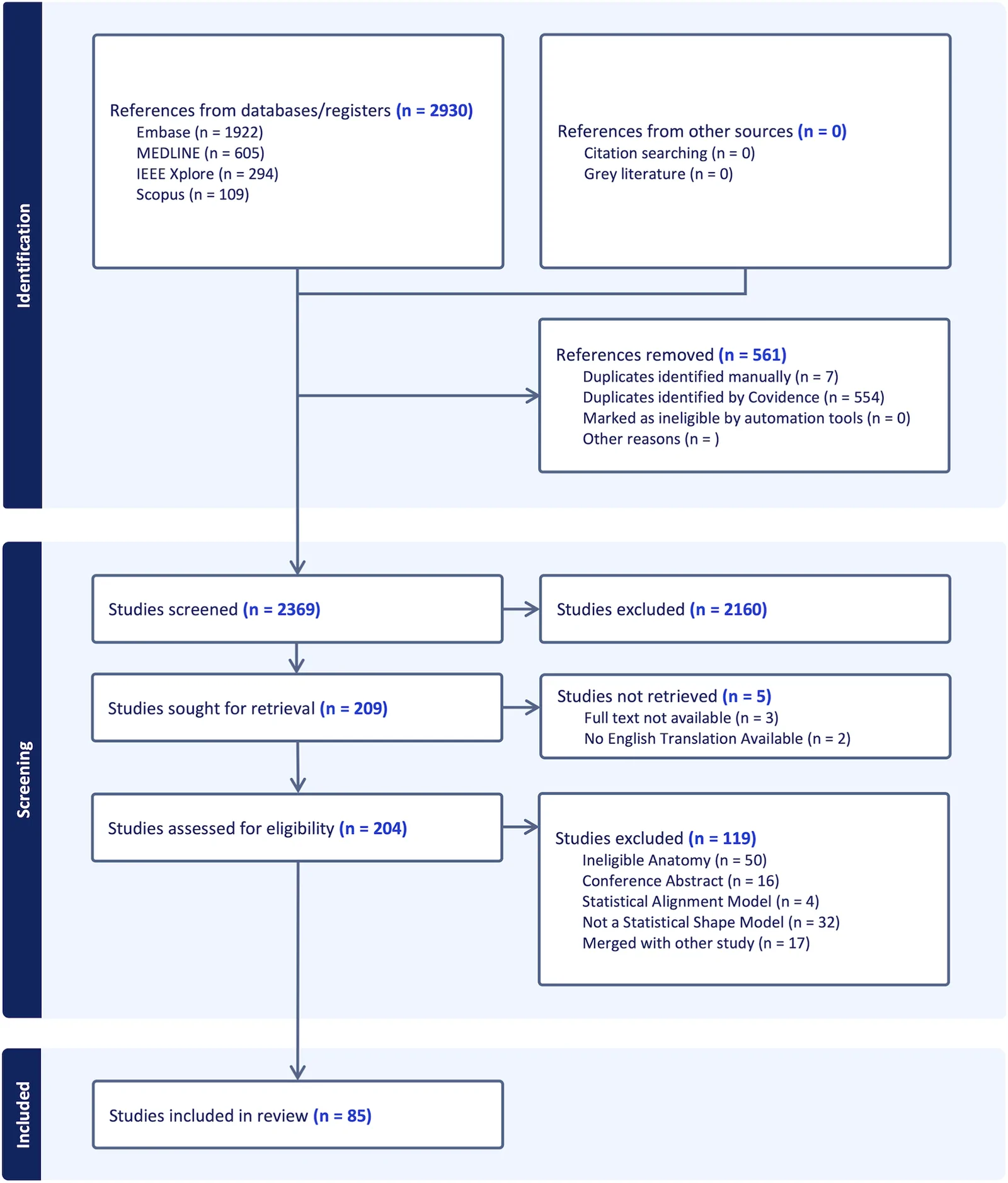
Scoping review selection process.

The final search strategy identified 2,930 records for review. Following duplicate removal, title and abstract screening, and full text screening, 102 records remained, of which 17 were combined into other records, resulting in 85 studies for data extraction. The full process is outlined in Figure 1. Details of study characteristics and the findings from individual sources of evidence are reported in the Supplementary Material.

Of the 85 studies included, 64 (75%) were published in peer-reviewed journals, and 21 (25%) were published as conference papers. Within these 85 studies, 129 unique SSMs were presented. Sixty (71%) studies presented a single SSM, with the remaining 69 models being described in 25 (29%) studies. Studies presenting more than one model either compared a novel modelling method with state-of-the-art approaches or examined how different vertebral level pooling strategies affected results.

### 3.1 Applications of statistical shape modelling

**FIGURE 2:**
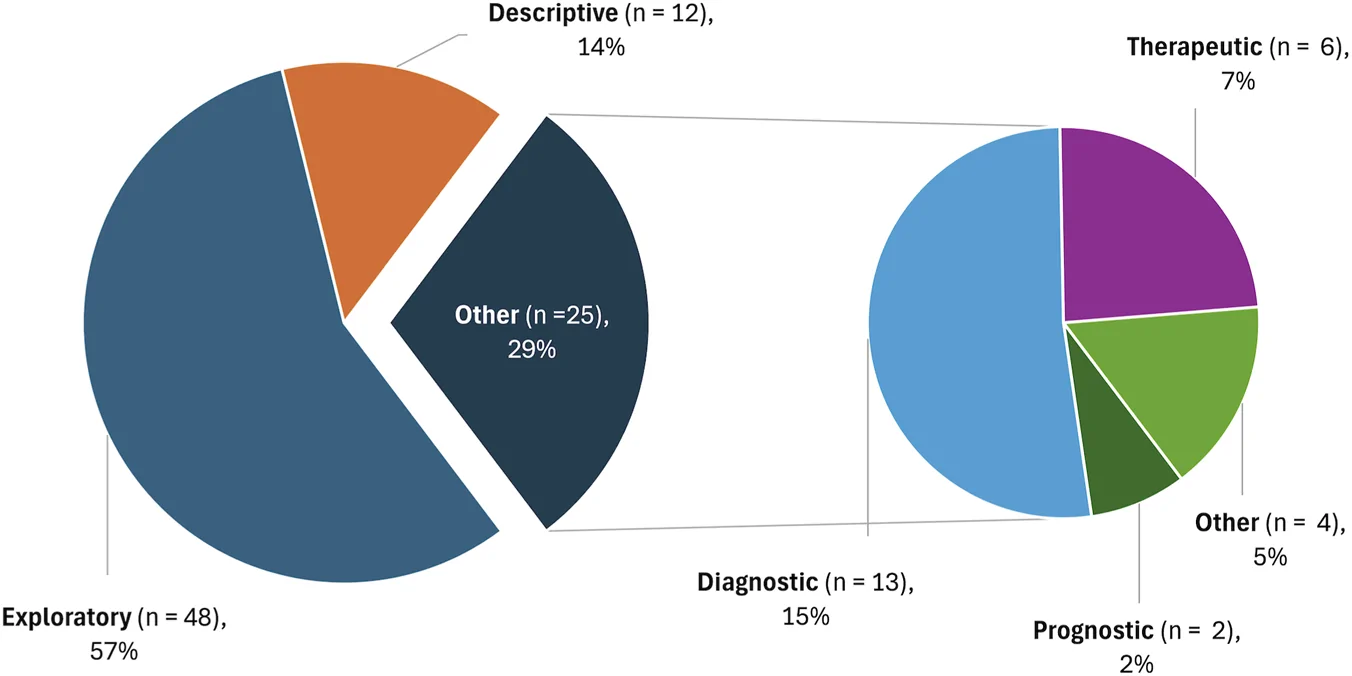
Proportion of studies coded for each application classification.

Most studies included were classified as either ‘exploratory’ (n = 48; 57%) or ‘descriptive’ (n = 12; 14%). Figure 2 shows the complete breakdown of the applications proposed. The four papers classified as ‘other’ presented datasets of finite element models created from SSMs as a research tool for use in biomechanical analysis.

Within the studies that went beyond exploratory or descriptive aims, common themes in the application of SSMs emerged. Prognostic applications comprised of vertebral fracture risk prediction; diagnostic applications involved fracture identification, IVD degeneration classification, and scoliosis classification; whilst therapeutic applications included surgical planning (e.g., pedicle screw guidance), and assistance for ultrasound-guided injections.

Three studies reported the use of SSMs in clinical populations, providing early evidence of feasibility. Möller et al. piloted MorphoXpress, a semi-automatic system for vertebral body fracture detection in German osteo-centres, benchmarking it against the current gold standard of radiologist review (Moller et al., 2011). Crimi et al. conducted a longitudinal case-control study to evaluate the effectiveness of a shape-based classifier in predicting vertebral fractures in postmenopausal women (Crimi et al., 2010). In another retrospective clinical study, Vijayan et al. used an ASM to develop and evaluate an automatic algorithm for surgical planning of pedicle screw trajectory (Vijayan et al., 2019). These studies begin to explore how SSMs might be applied in clinical contexts, but no studies have yet taken the step towards influencing practice directly.

### 3.2 Statistical shape modelling methods

#### 3.2.1 Landmarking and dimensionality reduction

Of the 85 studies presenting SSMs, 53 (62%) were three-dimensional (3D) and 32 (38%) were two-dimensional (2D). Table 3 shows a summary of the various landmarking methods used. Of the five studies coded as ‘other’, three did not state the method of landmark selection, and two used a method of statistical modelling that did not require landmarks to be selected. Automatic labelling methods were more common in 3D studies, where they accounted for 61% (n = 33) of models. Conversely, manual landmarking studies encompassed 68% (n = 21) of 2D studies. This was reflected in the number of landmark points to describe each vertebra, with 2D models ranging from four to 98, and 3D models ranging from 6 to 16,384.

**TABLE 3:** Methods used for selecting the landmarks for each study.

Landmarking method	Number of studies
Manual	30 (35%)
Semi-automatic	16 (19%)
Automatic	33 (39%)
Other	5 (6%)
Total	85 (100%)

Isolation of anatomy was often performed at the landmarking stage; of studies that isolated a region, 63% (n = 24) used manual landmark selection, and 18% (n = 7) used semi-automatic methods. Four (11%) studies used automatic selection of landmarks with custom algorithms. Table 4 shows the number of studies isolating each region of the vertebra.

**TABLE 4:** Studies isolating different vertebral regions (percentages do not sum to 100% as some studies modelled multiple regions).

Vertebral region modelled	Number of studies
No isolation/whole vertebra modelled	42 (54%)
Vertebral body	36 (46%)
Pedicles	9 (12%)
Posterior elements	1

Almost all studies that did not model the whole vertebra chose to model the vertebral body, with a further nine also modelling the pedicles. A single study isolated the vertebral posterior elements (Foltz et al., 2026). One paper by Hollenbeck et al. isolated the endplate of the S1 vertebra, however no detail was given on how the S1 endplate was defined (Hollenbeck et al., 2018).

Once landmarking and point-to-point correspondences had been established, classical PCA was the most common method of dimensionality reduction, encompassing 84% (n = 71) of included studies. Within the 14 (16%) studies not using classical PCA, eight were coded as exploratory studies presenting new methods of creating SSMs, including Principal Geodesic Analysis, Partial Least Squares Regression, Support Vector Machine-based approaches, Independent Component Analysis, and Multilevel Component Analysis.

#### 3.2.2 Vertebral levels and pooling

**FIGURE 3:**
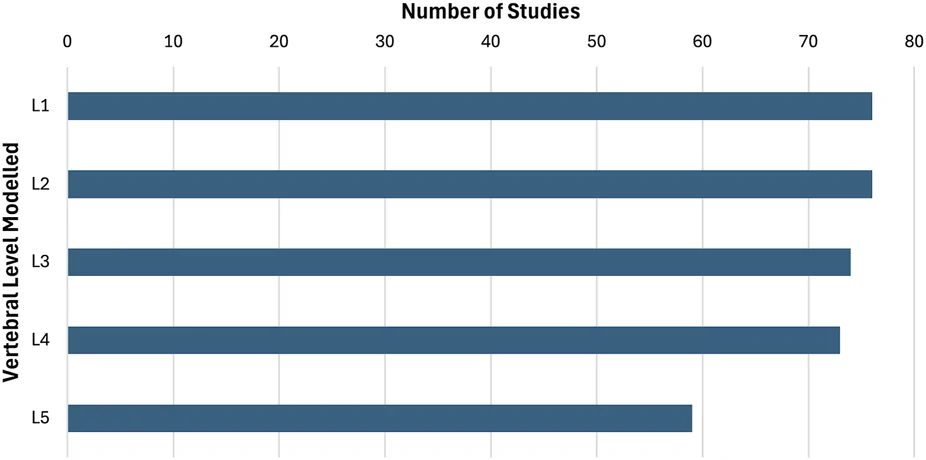
Frequency of each vertebral level modelled in included studies.

Figure 3 shows how commonly each vertebral level was modelled in the included studies. Fifty-seven (67%) of the identified studies, modelled the entire lumbar spine (L1 – L5). A small number of studies modelled an individual vertebral level (n = 8; 9%). The number of studies modelling L5 is markedly lower than the other lumbar levels, as many authors decided to exclude the L5 vertebra from their models, with 12 (14%) studies presenting an SSM analysing L1 – L4.

The L5 vertebra was also frequently excluded from studies that chose to pool vertebral levels. There were 28 (33%) studies that analysed multiple vertebral levels as one shape, allowing multiple vertebrae from each participant to contribute to the training data without increasing the number of participants. The most popular method was to pool all lumbar vertebrae as one shape (n = 9; 11%), however a significant portion chose to exclude L5 (n = 7; 8%). Table 5 shows the complete breakdown of the number of studies pooling vertebral levels.

**TABLE 5:** Pooled vertebral levels within included studies (values do not sum to 27 as some studies included multiple pools).

Pooled levels	Number of studies
T12 – L4	3
L1 – S1	1
L1 – L5	9
L1 – L4	7
L1 – L3	3
L2 – L3	1
L4 – L5	2
Not stated	3

#### 3.2.3 Single and multi-level methods

**FIGURE 4:**
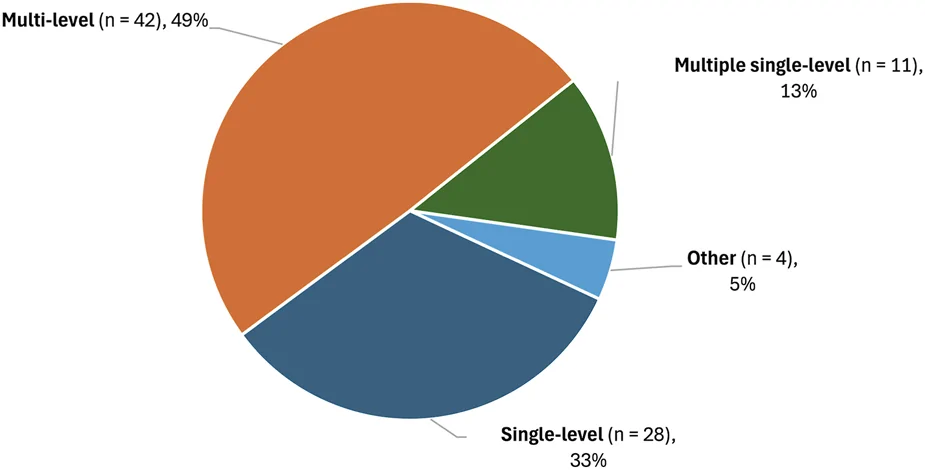
Distribution of studies by spinal modelling method.

Most studies included in this review modelled the spine as a multi-level object, with 42 (49%) studies conducting multi-level modelling and 11 (13%) others creating multiple single-level models. The number of studies using each modelling method is presented in Figure 4. Of the four studies coded as ‘other’, two presented both single-level and multi-level models for analysis, one pooled only adjacent vertebral levels, and one did not state the modelling methodology.

Many multi-level modelling methods have been proposed. For example, Neubert et al. created a multi-level SSM by first extracting a continuous 3D spine curve using fit to vertebral body and IVD centres, which captures global spinal alignment (Neubert et al., 2011). PCA is applied to this curve to capture the primary patterns of alignment variation. Individual SSMs of vertebral bodies and IVDs are then superimposed along the curve. Local models are deformed independently, but their placement is informed by the global spine shape. In contrast, Sciortino et al. created a model that treats the entire lumbar spine as one rigid shape (Sciortino et al., 2022). This approach is simpler but may conflate global and local sources of variation.

Some authors address the trade-off between modelling complexity and the influence of alignment by focusing on smaller spinal segments. For example, Caprara et al. constructed SSMs of Functional Spinal Units (FSUs), which comprise of adjacent vertebrae and the intervening IVD (Caprara et al., 2021). FSUs are small enough that vertebral alignment has a reduced impact on local shape variation but are still able to capture relationships across levels.

### 3.3 Populations studied

Two studies included in this review did not include information on the training population used (Wesarg et al., 2012; Amiranashvili et al., 2024). There was a large amount of heterogeneity in the reporting of the remaining 93 (98%) studies, making comprehensive analysis difficult. Furthermore, there was significant overlap in the populations, with some datasets being used in multiple studies, but also combined with additional data. As a result, no duplicate training populations were identified. In 31% (n = 26) of studies, there was no mention of a source for the training data. The common sources identified are presented in Table 6.

**TABLE 6:** Repeated sources of training data within included studies.

Source	Number of studies
Saint-Justine Hospital (Montréal, Canada)	6 (7%)
SpineWeb (Wang et al., 2021)	5 (6%)
VerSe: Large Scale Vertebrae Segmentation Challenge (Sekuboyina et al., 2021)	4 (5%)
National Center for Spinal Disorders (Budapest, Hungary)	2 (2%)
Kingston General Hospital (Kingston), Canada	2 (2%)
Vancouver General Hospital (Vancouver, Canada)	2 (2%)

There was a large amount of variance in the size of the training data in each study. The number of participants ranged from 3 to 2,520, and the number of vertebrae in the training data ranged from 2 to 15,120. Three (4%) reported only the number of vertebrae included. The mean number of participants and vertebrae in the training data was 153 and 1,179 respectively. Table 7 shows the full distribution of dataset size across all studies reported.

**TABLE 7:** Distribution of training and testing data size across reported studies.

Size of training data	Training data		Testing data	
	Number of studies -participants	Number of studies - vertebrae	Number of studies -participants	Number of studies - vertebrae
1–9	8 (11%)	2 (3%)	7 (21%)	2 (6%)
10–99	41 (55%)	24 (31%)	20 (61%)	11 (35%)
100–999	24 (32%)	35 (45%)	4 (12%)	11 (35%)
1,000–9,999	2 (3%)	15 (19%)	2 (6%)	7 (22%)
10,000–99,999	0 (0%)	2 (3%)	0 (0%)	0 (0%)
Total	75 (100%)	78 (100%)	33 (100%)	31 (100%)

Of the included studies, 34 (40%) tested the presented SSM on a unique testing population, whilst a further 23 (27%) employed some form of leave-out validation. On average, the 34 testing datasets were smaller than their corresponding training datasets, with a mean of 121 participants and 904 vertebrae.

Reporting of demographic characteristics for testing populations was limited and variable. For example, only 11 (12%) studies reported the age of the testing population, so these findings could not be generalised. For consistency, further demographic characteristics are reported for training datasets only.

**FIGURE 5:**
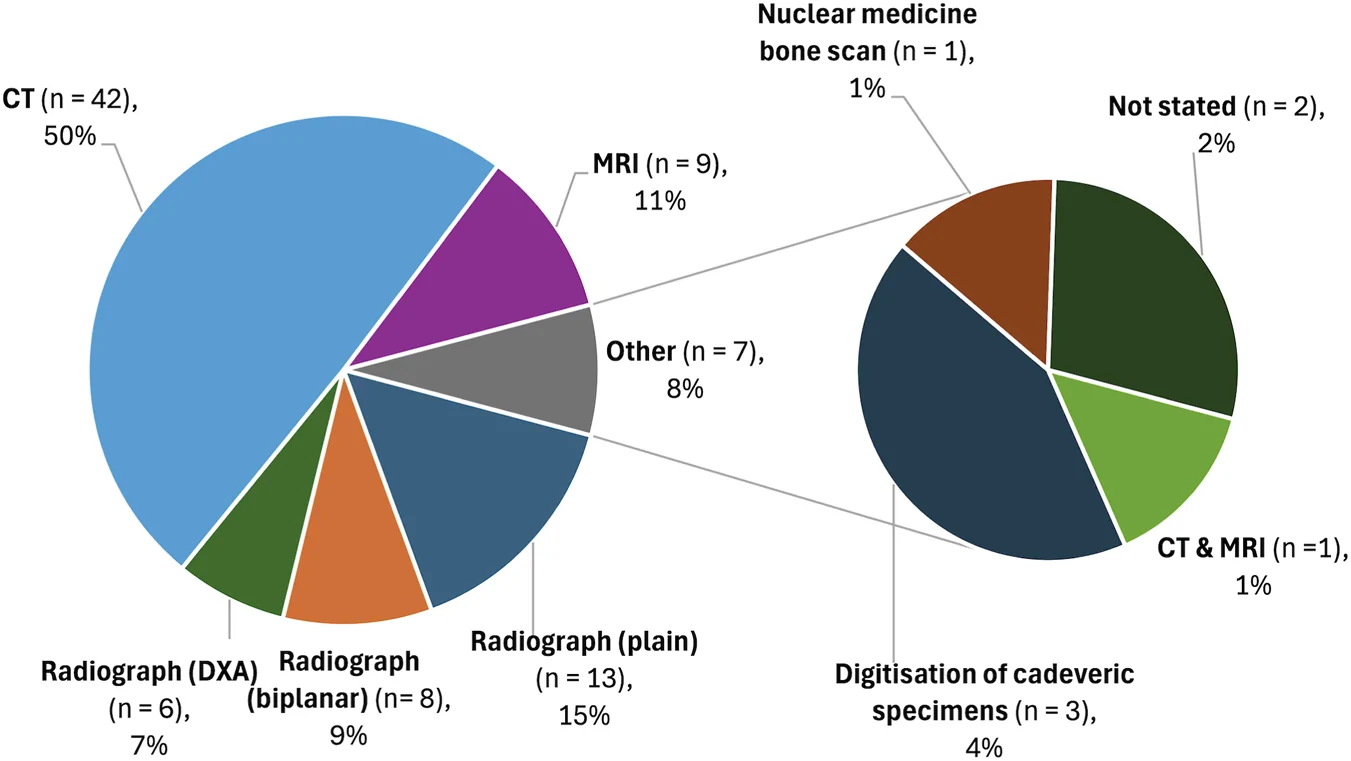
Imaging source modalities used in training datasets.

The most common imaging modality used as training data was computed tomography (CT), accounting for nearly half of the studies (n = 42; 50%). Figure 5 shows the full breakdown of imaging modalities seen in the review. Only one study used a combination of imaging modalities for the training data (Gilakjan et al., 2020).

**FIGURE 6:**
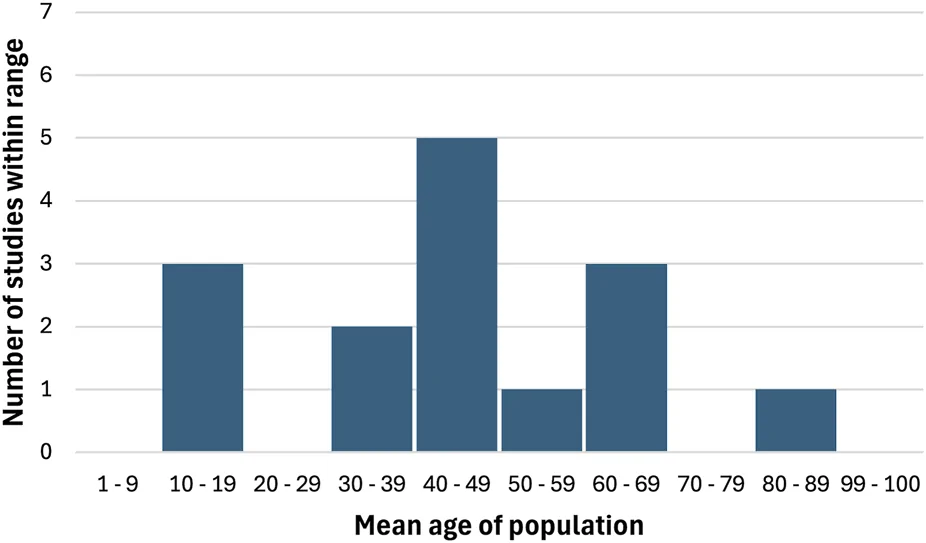
Imaging source modalities used in training datasets.

Age data were reported inconsistently across the included studies. The majority of the studies (n = 61; 72%) either did not report the age of the training population or reported the age data unclearly. Only 17 (20%) studies reported the mean age of the population. Figure 6 shows the distribution of mean ages reported within the training data.

Twenty-eight (33%) studies clearly reported the sex distribution of the training populations. The data was skewed towards the female population, with half of studies considering a predominantly female population, five studies considering only female participants, and three studies considering only male participants. Overall, about 58% (n = 1961) of the training data were of female subjects.

**FIGURE 7:**
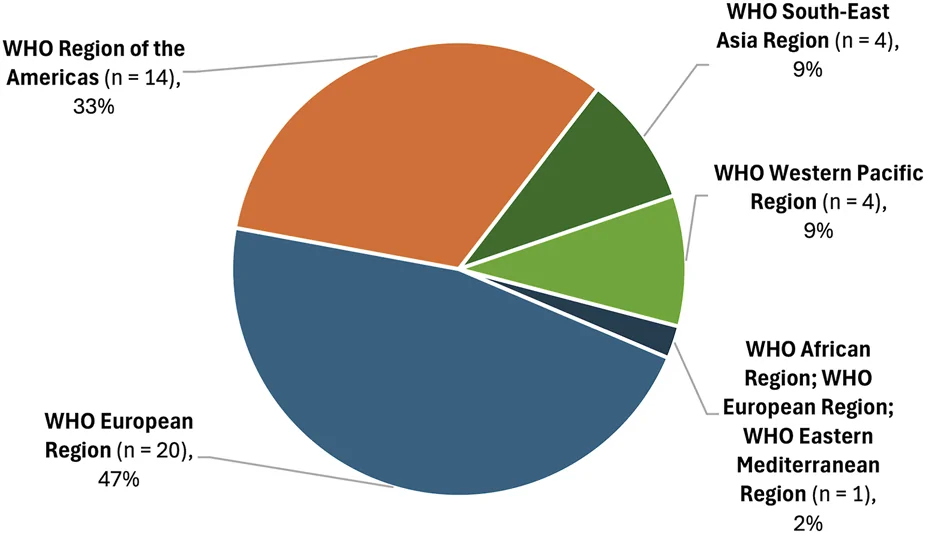
Distribution of training population by WHO region reported.

The training population region of origin was reported in 43 (51%) of the included studies. One study used training data across multiple WHO regions (Zlolniski et al., 2019). Figure 7 shows the full breakdown. The training data presented were heavily biased towards the Americas and Europe, with the two regions accounting for 80% of studies reporting the region of origin.

Training data primarily consisted of healthy populations, or populations with no reported pathology. Table 8 shows the number of studies investigating each condition.

**TABLE 8:** Distribution of spinal conditions included in training data across studies (percentages do not sum to 100% as some studies included multiple conditions).

Spinal condition	Number of studies
Healthy/No reported condition	62 (73%)
Trauma/Fracture	13 (15%)
Scoliosis	14 (16%)
Osteoporosis	7 (8%)
IVD degeneration/herniation	3 (4%)
Other: *Low back pain*	2 (2%)
Other: *Calcifications*	1 (1%)
Other: *Spinal canal stenosis*	2 (2%)
Other: *Pathological*	2 (2%)

### 3.4 Summary of SSM construction approaches by application

Table 9 aggregates the key construction and population characteristics of included SSMs by application category, providing a practical synthesis of the methodological approaches employed across different research and clinical aims. Exploratory and descriptive studies, which together account for the majority of included studies, are excluded from this summary as their primary aim was methodological development or population characterisation rather than a defined clinical or research application, and as such do not lend themselves to meaningful cross-tabulation of construction approach against aim. Given the limited number of studies within each category, the full breakdown of all approaches used is not reported, as the small absolute numbers are unlikely to be of practical value. The complete details for all studies are available in the Supplementary Material.

**TABLE 9:** Summary of dominant SSM construction approaches by clinical application category.

Application	Dimensions	Landmarking	Vertebral features	Dimensionality reduction	Vertebral pooling	Single vs. multi-level	Imaging modality
Diagnostic (n = 13)							
Scoliosis Classification (n = 5)	3D (n = 4/5)	Mixed	Whole vertebra (n = 3/5)	Classical PCA (n = 4/5)	No (n = 3/5)	Multi-level (n = 4/5)	Radiograph (biplanar) (n = 3/5)
Vertebral Fracture Detection (n = 4)	2D (n = 4/4)	Mixed	Mixed	Classical PCA (n = 4/4)	Mixed	Mixed	Mixed
IVD Degeneration Classification (n = 2)	Mixed	Mixed	Vertebral body (n = 2/2)	Classical PCA (n = 2/2)	No (n = 2/2)	Multi-level (n = 2/2)	MRI (n = 2/2)
Osteoporosis Detection (n = 1)	3D	Automatic	Whole vertebra	Classical PCA	Yes	Multiple pooled single-level	Not stated
Spinal Stenosis Classification (n = 1)	2D	Manual	Posterior elements	Classical PCA	No	No	MRI
Prognostic (n = 2)							
Vertebral Fracture Risk Assessment (n = 2)	2D (n = 2/2)	Manual (n = 2/2)	Vertebral body (n = 2/2)	Classical PCA (n = 2/2)	Mixed	Mixed	Radiograph plain (n = 2/2)
Therapeutic (n = 6)							
Pre-op Spinal Fusion Planning (n = 4)	3D (n = 4/4)	Automatic (n = 4/4)	Whole vertebra (n = 4/4)	Classical PCA (n = 4/4)	No (n = 3/4)	Mixed	CT (n = 3/4)
Image Guided Spinal Analgesia (n = 2)	3D (n = 2/2)	Automatic (n = 2/2)	Whole vertebra (n = 2/2)	Mixed	No (n = 2/2)	Mixed	CT (n = 2/2)

There is evidence that authors are already adapting their approaches in a clinically logical manner according to the intended application. Pre-operative spinal fusion planning studies consistently used 3D models and CT imaging, reflecting the need for high-resolution, volumetric anatomical detail to support surgical decision-making. Image-guided spinal analgesia studies similarly favoured 3D models, with CT as the dominant modality, consistent with the precision requirements of needle trajectory planning. Scoliosis classification studies predominantly employed multi-level modelling, which is an intuitive methodological choice given that scoliosis is fundamentally a multi-level deformity. These patterns suggest that the field is beginning to develop application-specific methodological norms, even in the absence of formal consensus guidelines.

However, several application categories show considerable methodological heterogeneity, reflected by the “Mixed” entries across multiple variables. This is particularly evident in vertebral fracture detection and vertebral fracture risk assessment, where no dominant approach emerges across dimensions, landmarking, pooling, or modelling strategy. Given the small number of studies within each category, it is not possible to draw firm conclusions about whether this variation reflects genuine methodological uncertainty or simply the limited evidence base available.

## 4 Discussion

This scoping review aimed to identify all studies that applied statistical shape modelling (SSM) to the human lumbar spine. Lumbar spine SSMs have been published across a range of journals, indicating ongoing interest in the field; however, no clear upward or downward trend in publication numbers can be discerned. The research community appears relatively focused, with many recurring authors contributing to multiple studies.

### 4.1 Applications of statistical shape modelling

The majority of the studies (71%) included in this review were exploratory or descriptive (Figure 2). Whilst they do not provide direct clinical outputs, they have been important in establishing how SSMs can be applied to the lumbar spine. These studies have supported the refinement of segmentation and registration methods, enabled reproducible quantification of morphological variation, and generated population reference models. It has also provided insight into patterns of spinal shape associated with age, sex, and pathology. Together, these studies have established the methodological basis of lumbar spine SSMs and created a platform for work directed towards clinical application.

However, the persistence of exploratory work as the dominant output raises questions about the pace of clinical translation. A large amount of work in the field appears to be iterating on methodology rather than building cumulatively towards defined clinical endpoints, suggesting that the lumbar spine SSM community is not yet unified around shared clinical priorities that would direct methodological development more purposefully.

As described in the Results, prognostic, diagnostic, and therapeutic applications were identified across the included studies, and three studies examined SSMs in genuine clinical contexts. None of the reviewed studies, however, demonstrated a direct change to clinical practice or impact on patient care. A common barrier to the clinical application of SSMs is the difficulty of accessing suitable patient populations. For example, Mustapha et al. present an ASM for more accurate segmentation of vertebrae to enable classification of fractures as part of a Content-Based Medical Image Retrieval (CBMIR) system (Mustapha et al., 2015). The system is technically advanced and supported by a robust testing methodology, utilising the publicly available NHANES II (Second National Health and Nutrition Examination Survey) database for validation. Whilst databases like NHANES II are useful for preliminary testing, clinical populations are crucial for confirming generalisability.

Several articles reported limitations in their training and testing data. Möller et al. identified issues with data collection, particularly the absence of blinding and the lack of consensus readings (Moller et al., 2011). Similarly, Crimi et al. noted data limitations, including the inability to assess the L5 vertebra within their dataset (Crimi et al., 2010). Vijayan et al. encountered problems with the quality of CT scans, which were insufficient for accurate analysis of the pedicle screw planning algorithm (Vijayan et al., 2019). The authors recommended CT slice thickness be no larger than 1–1.5 mm. However, such thin-slice acquisitions typically require higher radiation doses and longer scan times. Whilst this is generally achievable in preoperative scans, routine clinical spine CT is often reconstructed at thicker slice widths (up to ∼3 mm) (American College of Radiology, 2026). This becomes a significant limitation when retrospectively analysing non-dedicated studies (e.g., CT abdomen/pelvis) that were not acquired with the spine as the primary focus, which are commonly reconstructed at thicker slice widths (up to ∼5 mm) (American College of Radiology, 2022). The emergence of MRI-derived synthetic CT, in which CT-like bone visualisation is generated from MRI data, may offer a future solution to this imaging constraint (Costa et al., 2026).

Another limitation noted is the limited impact of proposed SSM applications on reducing clinical workload. For example, Möller et al. emphasise that vertebral deformities can have a wide range of differential diagnoses, and automated outputs still require interpretation by an experienced radiologist in their clinical context (Moller et al., 2011). Though the continued need for clinical expertise may undercut the utility of such tools, SSM tools still offer valuable support to radiologists by standardising measurements and highlighting subtle abnormalities, complementing their expert judgement. This may be particularly useful in busy departments or for less experienced readers.

Finally, the validation of these systems often depends on expert opinion as a reference standard, which may introduce some degree of subjectivity. However, approaches such as double reporting with consensus are widely accepted and provide a robust reference standard in imaging research. Comparisons with such standards may not fully capture clinical impact, and evaluation against patient outcomes could provide complementary evidence of clinical benefit. However, outcome-based validation can be challenging to interpret, particularly for conditions such as vertebral fractures that may heal over time, making it difficult to attribute patient outcomes directly to model performance. Nonetheless, such validation may not always be necessary for tools primarily intended to improve efficiency or standardisation.

### 4.2 Statistical shape modelling methods

#### 4.2.1 Isolation of anatomy

A variety of methods for SSM construction were presented. Most studies did not stray from the mathematical foundation originally proposed by Cootes et al. (1992). One of the main sources of variation lies in the training data and its preparation, such as landmarking methods. No clear consensus emerged on the most effective technique for this.

As shown in Table 9, the region of the vertebra modelled appears to reflect the clinical demands of the intended application. Therapeutic studies consistently modelled the whole vertebra, as surgical planning requires a complete representation of vertebral geometry including the posterior elements and pedicles. In contrast, diagnostic and prognostic studies more commonly isolated the vertebral body, reflecting its central role in the pathological processes being studied. This suggests that whilst no universal standard exists for anatomical isolation, the field is making methodologically rational choices at the level of individual application categories.

Methods for defining the boundaries of the vertebral body were inconsistently reported and usually performed manually. The manual definition of vertebral body boundaries introduces operator-dependent variability that is rarely quantified or reported, yet is likely to have a non-trivial impact on the resulting shape model. Some authors gave no information whereas others, such as Boisvert et al., clearly reported the vertebral body landmarks selected (Boisvert et al., 2008a; 2008b). Pereañez et al. modelled the entire vertebra but used statistical shape decomposition to split the vertebrae into different subregions for analysis, offering a more objective method of isolation (Pereanez et al., 2015).

#### 4.2.2 Vertebral levels and pooling

As shown in Table 9, pooling decisions across diagnostic and prognostic categories appear inconsistent, and the limited justification provided in many studies makes it difficult to assess whether these decisions were driven by clear methodological or clinical reasoning. In contrast, pooling is avoided in therapeutic applications such as pre-operative spinal fusion planning, where individual vertebral geometry is essential for accurate surgical decision-making. More explicit reporting of the rationale for pooling decisions in future studies would improve the interpretability and comparability of results.

The most clinically significant pooling decision identified across the literature is the frequent exclusion of L5. L5 is classified as an atypical vertebra, differing anatomically from the other lumbar levels with a shorter, broader vertebral body and a wedge shape contributing to the lumbosacral angle (Gray, 2016). In addition, L5 may present as a lumbosacral transitional vertebra, most commonly through partial or complete sacralisation (Fang et al., 2025). Since L5 is also more frequently associated with spinal pathologies such as disc herniation and spondylolysis, it is often analysed separately (Kjaer et al., 2005; Foreman et al., 2013). These variations may reduce model coherence. For example, Gooya et al. showed that the model evidence of their SSM was maximised when L1 – L4 were clustered separately from L5 (Gooya et al., 2018).

#### 4.2.3 Single and multi-level modelling

Exactly half of the studies considered vertebral alignment as a multi-level model, but no overall consensus emerged on a gold standard approach. However, the choice of modelling approach does appear to reflect the clinical demands of the intended application, with multi-level modelling dominating scoliosis classification studies where vertebral alignment across levels is central to the diagnosis, and single-level approaches more common in diagnostic studies where localised vertebral morphology is the primary focus (Table 9). This suggests that whilst field-wide methodological consensus remains absent, application-specific norms are beginning to emerge organically. This is particularly logical for therapeutic applications, where a model that conflates global spinal alignment with local vertebral morphology could directly compromise surgical planning accuracy. Whether the same methodological distinctions carry equivalent clinical significance for diagnostic and prognostic applications remains unclear, and represents an important area for future investigation.

### 4.3 Population studies

A major limitation identified was the inconsistency of population reporting. Details such as age and sex distribution were often missing or ambiguously presented. Without standardised reporting of dataset characteristics, it becomes challenging to interpret performance metrics or replicate modelling pipelines.

Even amongst studies that used shared datasets, reporting was inconsistent. Some studies reused well-known public datasets but often used only a subset of the available data and did not specify which samples were included (Gilakjan et al., 2020; Sekuboyina et al., 2021). This makes it difficult to assess potential overlap between studies. The ambiguity surrounding dataset composition highlights the need for clearer data reporting standards. Hollenbeck et al. provide a good example of clear training data reporting, specifying the source, size, and demographic breakdown of their dataset (Hollenbeck et al., 2018).

Access to quality data may be a general challenge in this field. Studies appear to rely on the datasets that are most readily available to the authors, rather than gathering data that aligns with their research objectives. This approach is practical but can limit data diversity and introduce biases. Consequently, the models developed might not fully capture the range of anatomical variations present in broader populations.

This is evident in the skewed demographic distribution of the training data, which is heavily biased towards America and Europe. The underrepresentation of other regions limits the generalisability of the models to global populations, particularly given evidence that vertebral morphology varies across human populations (Zlolniski et al., 2019).

The demographic skew towards female participants is likely driven by the higher prevalence of conditions such as osteoporosis and fractures, which are more common in women (Lorentzon et al., 2022; Willers et al., 2022). Whilst this may reflect the pathologies studied, it suggests that the models might not fully account for the anatomical differences that are unique to male populations (Bailey et al., 2016).

### 4.4 Recommendations for future research

Future studies should ensure that training data is carefully aligned with the clinical question under investigation. Researchers should select or curate data that is anatomically and demographically representative of the target population. This includes ensuring an appropriate sample size. Prior work has suggested that approximately 200 samples are required for reliable model training (Audenaert et al., 2019). More deliberate gathering of training data is also important with respect to resolution. As demonstrated by Vijayan et al., some scans lacked sufficient resolution for presurgical planning, showing that SSMs cannot simply be created from readily available data but must be trained on imaging that matches the intended use case (Vijayan et al., 2019). Rather than standardising imaging protocols across the field as a whole, standardisation is likely to be most valuable within multicentre initiatives addressing a shared clinical question, where common protocols would allow data to be pooled across sites without compromising the alignment between training data and clinical purpose. Such coordinated, question-specific data collection would help address the sample size and demographic representativeness limitations identified in this review.

Improved consistency in reporting is also essential. This includes full documentation of population characteristics and model validation metrics, specifically compactness (the efficiency with which the model describes shape variation using few components), generalisability (its ability to represent shapes not included in the training data), and specificity (the extent to which shapes generated by the model resemble anatomically plausible vertebra). Without these, systematic reviews and meta-analyses cannot draw broader conclusions about model performance or clinical utility. To support the implementation of these recommendations, Table 10 outlines a proposed minimum reporting standard for future lumbar spine SSM studies.

**TABLE 10:** Proposed minimum reporting standard for future lumbar spine SSM studies.

Domain	Recommended reporting items
Training and testing populations	Sample size, age, sex distribution, WHO region, pathology, and imaging source for both populations; specific sample IDs in Supplementary Material where a dataset subset is used; performance metrics for the testing population
Imaging	Modality, slice thickness, reconstruction protocol
Correspondence method	Landmarking approach (manual, semi-automatic, automatic), number of landmarks, and method used to define landmarks or anatomical boundaries
Registration and alignment	Algorithm used for shape alignment or dense correspondence (e.g., GPA, ICP, non-rigid registration), software/toolkit, and pre-processing steps (e.g., scaling, pose normalisation)
Dimensionality reduction	Statistical framework (e.g., classical PCA, kernel PCA), number of components retained, variance explained
Vertebral levels	Levels included/excluded, with justification (e.g., L5 exclusion)
Multi-level modelling	Single-level, multi-level, or pooled approach; if pooled, which levels and rationale; if multi-level, method used to capture inter-vertebral relationships
Validation	Metrics reported (compactness, generalisability, specificity, reconstruction error, surface distance, landmark error), with acceptability thresholds

SSMs have shown promising potential in applications such as predicting vertebral fracture risk, surgical planning, and spinal deformity classification. Translation into clinical decision-making has not yet been achieved, largely due to challenges such as small sample sizes and methodological inconsistencies. As model performance depends on training data, strengthening datasets and standardising methods will help move the use of SSMs closer to clinical practice. Table 9 provides early indications of emerging methodological convergence within certain application categories, though the limited number of studies per category means that firm conclusions cannot yet be drawn.

At present, the most constructive role for SSMs may lie in exploratory analyses of clinical populations. By capturing patterns of anatomical variation that are not detected by conventional measurements, these models can generate new insights and hypotheses for testing through established research methods. In this way, SSMs are best understood as research tools for uncovering patterns of spinal variation that can be tested and refined in clinically focused studies. Moving forward, the greatest progress is likely to come from studies that focus on clinical populations and are designed around clear clinical questions, ensuring model development is aligned with practical needs in patient care.

Alongside this clinical focus, there is an opportunity to further integrate lumbar spine SSMs with deep learning. A small number of included studies already demonstrate hybrid approaches, retaining the SSM as an anatomically plausible shape prior whilst using deep learning components to improve automation and adaptability to challenging imaging conditions. Expanding this hybrid model offers considerable potential to clinical applicability, and represents a valuable direction for future research.

### 4.5 Limitations

This review has several limitations. Firstly, there was no second reviewer, which could have reduced errors and bias. At the study selection stage, a single reviewer introduces the risk of incorrectly including or excluding studies, potentially skewing the breadth of the review. At the data extraction stage, a single reviewer may introduce inconsistencies in how data are recorded and interpreted across studies, particularly given the heterogeneity of reporting identified throughout this review. Additionally, no grey literature search or citation scraping was conducted after extracting records from the databases, which may have resulted in the omission of relevant studies. However, to mitigate the limitations of single-reviewer screening, all inclusion decisions were regularly cross-checked against the predefined criteria. Furthermore, the use of predefined eligibility criteria and multiple databases likely mitigated some of these limitations.

Heterogeneity in reporting has been discussed at length for training data demographics, but similar inconsistencies occur in other study characteristics, making it challenging to compare model validation. The metrics reported varied substantially depending on the SSM constructed, its proposed application, and the reporting choices of individual authors, rendering systematic comparison of validation outcomes across studies not possible. Commonly reported measures included compactness, generalisability, specificity, reconstruction error, surface distance, and landmark error, though these were applied inconsistently and rarely accompanied by acceptability thresholds. Furthermore, as the complexity of SSM-integrated pipelines has increased, many studies have omitted model-level validation entirely in favour of application-specific performance measures. This inconsistency represents a significant methodological gap in the field and an area requiring standardisation in future research.

Similarly, whilst the source of training data was recorded as free text during extraction, this did not anticipate the level of detail needed to classify datasets by both origin (e.g., open-access versus institutional or opportunistically gathered) and type (e.g., raw imaging versus secondary or derived data such as segmentations or finite element models), representing a useful addition for future reviews in this area.

Finally, it should be noted that this review encompasses only academic literature. Commercial applications may be underrepresented, as these systems often disclose limited methodological details due to intellectual property constraints. Möller et al. employed a commercial system (MorphoXpress) but provided minimal information on the training data and the SSM creation process (Moller et al., 2011). It is likely that other commercial models withhold methodological details, hence complicating cross-study comparisons.

## 5 Conclusion

The current literature on lumbar spine SSMs is largely exploratory, combining analyses of shape variation with segmentation, registration, and landmarking, with the majority of published models representing methodological contributions or population-level morphological characterisations rather than clinically directed investigations. Whilst prognostic, diagnostic, and therapeutic applications have been proposed, none of the reviewed studies demonstrated a direct change to clinical practice or impact on patient care, and clinical evaluation of the method remains in its infancy.

Methodologically, classical PCA applied to landmark-based correspondence remains the dominant construction approach, though the unique challenges posed by the multi-level, mechanically coupled nature of the lumbar spine, including multi-level modelling strategy, vertebral level pooling, and anatomical boundary definition, remain without consensus.

Training datasets are predominantly drawn from Western, female-skewed populations, and inconsistent validation reporting across the literature precludes systematic comparison of model performance. Clearer reporting, consistent validation, and training data aligned with clinical questions and imaging standards will therefore be essential to accelerate progress. With these steps, lumbar spine SSMs have the potential to move beyond descriptive work and towards a technology that can support future clinical translation.

## Data Availability

The original contributions presented in the study are included in the article/Supplementary Material, further inquiries can be directed to the corresponding author.

## Footnotes

1 The WHO groups its 194 Member States into six regions for administrative and reporting purposes: the African Region, the Region of the Americas, the South-East Asia Region, the European Region, the Eastern Mediterranean Region, and the Western Pacific Region. These groupings are organisational rather than strictly geographical.
