# Supplementary material for: Statistical shape modelling of the human lumbar spine: a scoping review characterising the research landscape and potential clinical applications
Source: Front Bioeng Biotechnol. 2026 Sep 9;14:1884249. doi: 10.3389/fbioe.2026.1884249 (PMC13599120; doi:10.3389/fbioe.2026.1884249)
Supplement: Supplementary file 1 [file Table1.docx]

Applied search algorithms for a systematic scoping review on statistical shape modelling of the lumbar spine

| **Source** | **Search** | **Records Retrieved** |
| --- | --- | --- |
| MEDLINE (Ovid) | 1. (statistical adj3 (shape or shape+pose or finite element or morphometric or geometric) adj2 (model* or analys*)).mp.  2. ((Articulated or active) adj3 (Shape or Appearance) adj2 (Model* or analys*)).mp.  3. Point Distribution Model*.mp.  4. Active Appearance Model*.mp.  5. Principal Component Analysis/  6. Procrustes Analys?.mp.  7. (registration and segmentation).mp.  8. statistical model*.mp.  9. 7 and 8  10. 1 or 2 or 3 or 4 or 5 or 6 or 9  11. spine/ or exp cervical vertebrae/ or exp intervertebral disc/ or lumbar vertebrae/ or exp spinal canal/ or thoracic vertebrae/ or vertebral body/  12. (spine* or spinal).mp.  13. vertebra*.mp.  14. Intervertebra*.mp.  15. pedicle.mp.  16. endplate.mp.  17. 11 or 12 or 13 or 14 or 15 or 16  18. 10 and 17 | 605 |
| Embase (Ovid) | See MEDLINE(Ovid) | 1922 |
| IEEE Xplore | ((("statistical" NEAR/3 ("shape" OR "shape+pose" OR "finite element" OR "morphometric" OR "geometric") NEAR/2 ("model*" OR "analys*")) OR (("articulated" OR "active") NEAR/3 ("shape" OR "appearance") NEAR/2 ("model*" OR "analys*")) OR "Point Distribution Model*" OR "Active Appearance Model*" OR "Principal Component Analysis" OR "Procrustes Analys*” OR ("registration" AND "segmentation" AND "statistical model")) AND (("spine*" OR "spinal") OR "vertebra*" OR "intervertebra*" OR "pedicle" OR "endplate")) | 294 |
| Scopus | ( ( "statistical" near/3 ( "shape" OR "shape+pose" OR "finite element" OR "morphometric" OR "geometric" ) near/2 ( "model*" OR "analys*" ) ) OR ( ( "articulated" OR "active" ) near/3 ( "shape" OR "appearance" ) near/2 ( "model*" OR "analys*" ) ) OR "Point Distribution Model*" OR "Active Appearance Model*" OR "Principal Component Analysis" OR "Procrustes Analys?" OR ( "registration" AND "segmentation" ) AND ( "statistical model" ) )AND(("spine*" OR "spinal") OR "vertebra*" OR "intervertebra*" OR "pedicle" OR "endplate") | 109 |
