# Supplementary material for: Statistical shape modelling of the human lumbar spine: a scoping review characterising the research landscape and potential clinical applications
Source: Front Bioeng Biotechnol. 2026 Sep 9;14:1884249. doi: 10.3389/fbioe.2026.1884249 (PMC13599120; doi:10.3389/fbioe.2026.1884249)
Supplement: Supplementary file 2 [file Table2.docx]

Data extraction form used for systematic review

| **Field** | **Description and Instructions** |
| --- | --- |
| **General Information** | |
| ID | Unique ID number for all studies |
| Title | Title of article |
| Journal | Journal or conference title |
| DOI | Digital object identifier code |
| Publication date | Date of first publication, including online |
| Authors | All authors in “Last name, First name” format – Each new author on a new line |
| **Characteristics of Included Studies** | |
| Aim of Study | One-sentence summary of the aims of the model |
| Application of the model | Select all that apply:   - Exploratory - Descriptive - Diagnostic - Therapeutic - Prognostic - Other |
| **Methods** | |
| Number of models created | Number of models presented in this article |
| ***Training Population Characteristics*** | |
| Size of training population (participants) | State the number of participants used to train the model |
| Size of training data (vertebrae) | State the total number of vertebrae used to train the model |
| Training data image modality | Select all that apply:   - Radiograph (plain) - Radiograph (DXA) - Radiograph (biplanar) - CT - Ultrasound - MRI - Other |
| Sex distribution | Number of male / female / other / not stated |
| Age | Mean / median / IQR / SD / min / max |
| World Health Organisation region | Select all that apply:   - WHO African Region - WHO Region of the Americas - WHO South-East Asia Region - WHO European Region - WHO Eastern Mediterranean Region - WHO Western Pacific Region - Not reported |
| Conditions modelled | Select all that apply:   - Healthy / no condition stated - Osteoporosis - Scoliosis - Spondolysis / spondylolisthesis - IVD degeneration - Trauma / fracture - Other |
| Training population notes | Brief description of population and source used for training the model |
| ***Modelling*** | |
| Modelling dimensionality | - 2D - 3D |
| Levels modelled | Select all levels of the spine that were modelled:   - L1 - L2 - L3 - L4 - L5 |
| Vertebral features modelled | Select all that apply - If whole vertebra modelled select ONLY "whole vertebra":   - Endplate - Vertebral body - Pedicle - Lamina - Spinous process - Transverse process - Whole vertebra - Other |
| Vertebral feature isolation (description) | Briefly describe the method of isolation if only part of the vertebral body was modelled |
| Pooled vertebrae | Select yes/no, based on whether multiple vertebral levels were modelled as one vertebra:   - Yes - No - Other |
| Pooled vertebrae (description) | State how the vertebrae were pooled during modelling |
| Single- or multi-level model | - Single-level - Multi-level - Other |
| Multi-level modelling (description) | If multi-level modelling used, describe how the multi-level model was created |
| Labelling method | Select the method of point labelling used   - Manual - Semi-automatic - Automatic - Other |
| Labelling method notes | Brief description of labelling method |
| Number of landmarks per vertebra | Input the number of landmarks used for each vertebra to create the model |
| Number of landmarks per model | Input the total number of landmarks used to create the model |
| Dimensionality reduction method | Select method used for dimensionality reduction   - Classical PCA - Other |
| **Validation / Testing / Application** | |
| Validation method | Select all that apply   - Compactness - Generalisation and specificity errors - Application accuracy - Other |
| Application description | Brief description of the application proposed |
| Testing | Was the application proposed tested against a unique data set? – If no, skip to **Limitations**   - Yes - No |
| ***Testing Population Characteristics*** | |
| Size of testing population (participants) | State the number of participants that the application was tested on |
| Size of testing data (vertebrae) | State the total number of vertebrae that the application was tested on |
| Testing data image modality | Select all that apply   - Radiograph (plain) - Radiograph (DXA) - Radiograph (biplanar) - CT - Ultrasound - MRI - Other |
| Sex distribution | Number of male / female / other / not stated |
| Age | Mean / median / IQR / SD / min / max |
| World Health Organisation region | Select all that apply:   - WHO African Region - WHO Region of the Americas - WHO South-East Asia Region - WHO European Region - WHO Eastern Mediterranean Region - WHO Western Pacific Region - Not reported |
| Conditions present in testing data | Select all that apply:   - Healthy / no condition stated - Osteoporosis - Scoliosis - Spondylosis / spondylolisthesis - IVD degeneration - Trauma / fracture - Other |
| Testing population notes | Brief description of population and source used for testing the model |
| **Other Details** | |
| Study funding sources | State any sources of funding for the research |
| Conflicts of interest | State any possible conflicts of interest for the study authors |
